# Platycodin D inhibits the proliferation and migration of hypertrophic scar-derived fibroblasts and promotes apoptosis through a caspase-dependent pathway

**DOI:** 10.1007/s00403-022-02513-1

**Published:** 2022-12-16

**Authors:** Zhencheng Yu, Yun Li, Rao Fu, Yaxin Xue, Danyang Zhao, Dong Han

**Affiliations:** 1 Department of Plastic and Reconstructive Surgery, Shanghai Ninth People’s Hospital, Shanghai Jiao Tong University School of Medicine, 639 Zhizaoju Road, Shanghai, 200011 China; 2 Department of Burns and Plastic Surgery, Xiangya Hospital, Central South University, No. 87, Xiangya Road, Changsha, 410008 Hunan China

**Keywords:** Platycodin D, Hypertrophic scar, Extracellular matrix, Fibroblasts, Apoptosis

## Abstract

Abnormal fibroblast proliferation and excessive extracellular matrix (ECM) deposition lead to the formation of hypertrophic scars (HSs). However, there is no satisfactory method to inhibit the occurrence and development of HSs. In our study, platycodin D (PD), a natural compound extracted from *Platycodon grandiflorus*, inhibited HSs formation both in vitro and in vivo. First, qRT-PCR and Western blot were used to confirm PD dose-dependently downregulated the expression of Col I, Col III and α-SMA in human hypertrophic scar-derived fibroblasts (HSFs) (*p* < 0.05). Second, cck-8, transwell and wound healing assays verified PD suppressed the proliferation (*p* < 0.05) and migration of HSFs (*p* < 0.05), and inhibited the differentiation of HSFs into myofibroblasts. Moreover, PD-induced HSFs apoptosis were analyzed by flow cytometry and the apoptosis was activated through a caspase-dependent pathway. The rabbit ear scar model was used to further confirm the inhibitory effect of PD on collagen and α-SMA deposition. Finally, Western blot analysis showed that PD reduced TGF-β RI expression (*p* < 0.05) and affected matrix metalloproteinase 2 (MMP2) protein levels (*p* < 0.05). In conclusion, our study showed that PD inhibited the proliferation and migration of HSFs by inhibiting fibrosis-related molecules and promoting apoptosis via a caspase-dependent pathway. The TGF-β/Smad pathway also mediated the inhibition of HSFs proliferation and HSFs differentiation into myofibroblasts. Therefore, PD is a potential therapeutic agent for HSs and other fibrotic diseases.

## Introduction

Hypertrophic scars (HSs) are formed due to abnormal fibroblast proliferation and excessive collagen deposition during the healing process after trauma, bites, acne or surgeries [25]. The continuous growth of HSs is generally accompanied by pain and itching, resulting in an unsightly appearance, dysfunction and psychological stress in patients [7, 29]. Currently, the clinical treatment for HSs is mainly based on the combination of hormone drug injection, laser therapy and surgical radiotherapy [3, 14, 23]. However, due to the complicated pathogenesis of HSs, the effects of such treatments are not satisfactory, and the recurrence rate is still high [9, 14]. Therefore, it is necessary to perform further research to identify new and effective drugs.

**Fig. 1:**
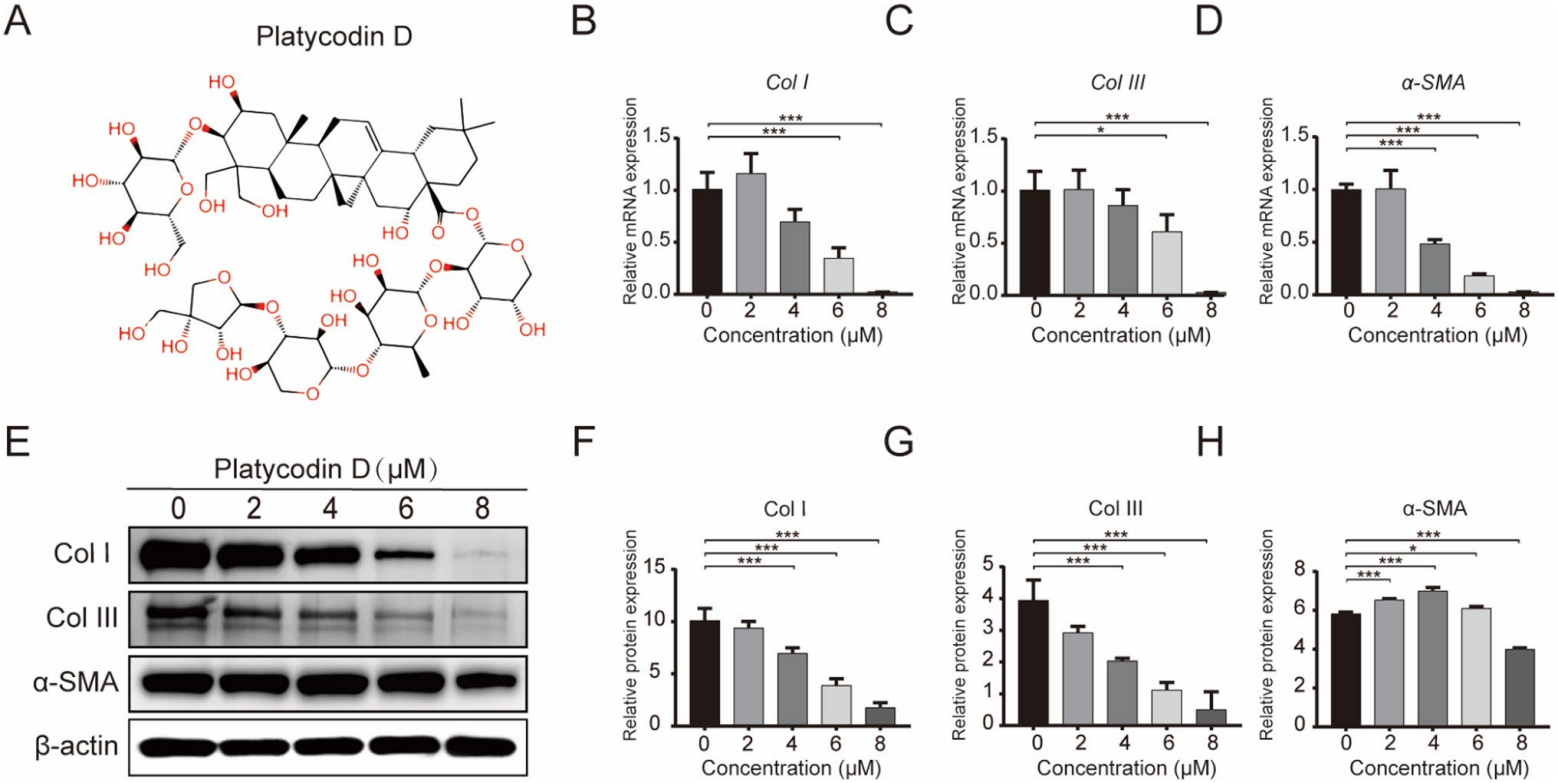
Effects of PD on the mRNA and protein levels of fibrosis-related molecules (**A**) Chemical formula of PD (C57H92O28; MW 1225). **B**–**D** qRT-PCR analysis of the mRNA levels of Col I, Col III and α-SMA after treatment with 0, 2, 4, 6 or 8 μM PD for 48 h. GAPDH served as the control. **E** Western blot analysis of the protein levels of Col I, Col III and α-SMA after treatment with 0, 2, 4, 6 or 8 μM PD for 48 h. β-actin served as the control. **F–H** Quantification of the protein levels in **E**, which were normalized to the level of β-actin. Each bar represents the mean ± SD. (*n* = 3). *p < 0.05, **p < 0.01 and ***p < 0.001

Platycodin D (PD) is a triterpenoid monomer compound isolated from the *Platycodon grandiflorum* plant (Fig. 1A). According to previous studies, PD has anti-inflammatory effects [28, 32] and can induce apoptosis [35], alleviate liver fibrosis [22] and reverse hypertrophy and fibrosis in myocardial cells [20]. In addition, PD exerts anti-tumor effects through biological activities [26], such as inhibiting cell proliferation and migration [30], arresting the cell cycle [13] and activating autophagy [16, 33]. However, the effect of PD on the proliferation and apoptosis of hypertrophic scar-derived fibroblasts (HSFs) is not yet clear. Therefore, this study aimed to explore the effects of PD on HSs and determine the potential molecular mechanism to provide new strategies and targets for the treatment of HSs.

## Materials and methods

### HSFs isolation and expansion

Hypertrophic scar (HS) samples were collected from patients of the Department of Plastic and Reconstructive Surgery, Shanghai Ninth People’s Hospital, and written consent was provided. None of the patients had received treatments before surgery. The subcutaneous fat tissue was removed, and the HSs samples were immersed in 0.25% dispase II (Roche, Germany) at 4 °C overnight. The dermis was then separated from the epidermis and digested with 0.25% collagenase I (Sigma, USA) at 37 °C for 4–6 h. The isolated HSFs were cultured in complete high glucose Dulbecco’s modified Eagle’s medium (DMEM, Gibco, USA) containing 10% fetal bovine serum (FBS, Gibco, USA), 100 U/mL penicillin and 100 μg/mL streptomycin (Gibco, USA). Cells from the third passages (P3) were used in all experiments [18].

### Treatment of HSFs

HSFs were seeded in 6-well plates at a concentration of 1 × 10^5^/mL in 2 mL of complete medium (CM) per well. A total of 15 wells were divided into five groups (*n* = 3), and each group was treated with 0, 2, 4, 6 or 8 μM PD. A total of 18 wells were divided into six groups (*n* = 3), and each group was treated with PD (0 μM), TGF-β1 (5 ng/mL), TGF-β1 (5 ng/mL) + PD (2 μM), TGF-β1 (5 ng/mL) + PD (4 μΜ), TGF-β1 (5 ng/mL) + PD (6 μM) or TGF-β1 (5 ng/mL) + PD (8 μΜ). PD was purchased from Yuanye Bio-Technology (Shanghai, China) and dissolved in ddH_2_O to prepare a 10 mM stock solution. Human TGF-β1 (Peprotech, USA) was diluted to prepare a 20 ng/mL stock solution.

### Quantitative real-time PCR (qRT-PCR)

HSFs were treated with different concentrations of PD for 24 h and lysed with TRIzol reagent (Invitrogen, CA, USA), and total RNA was extracted and purified. cDNA was obtained by reverse transcription of 1000 ng of RNA. The sequences of the primers used in the present study were as follows: *ColA1* forward: 5'-GAGCGGTAACAAGGGTGAGC-3', reverse: 5'-CGGTGGTTTCTTGGTCGGT-3'; *Col3A1* forward: 5'-TTGAAGGAGGATGTTCCCATCT-3', reverse: 5'-ACAGACACATATTTGGCATGGTT-3'; *α-SMA* forward: 5'-GTGTTGCCCCTGAAGAGCAT-3', reverse: 5'-GCTGGGACATTGAAAGTCTCA-3'; *TGF-β1* forward: 5'-GGCCAGATCCTGTCCAAGC-3', reverse: 5'-GTGGGTTTCCACCATTAGCAC-3'; *GAPDH* forward: 5'-ACAACTTTGGTATCGTGGAAGG-3', reverse: 5'-GCCATCACGCCACAGTTTC-3'. The gene expression levels of *Col I, Col III* and α-smooth muscle actin (*α-SMA*) were determined using the TB GREEN Premix Ex Taq^™^ kit (Takara, Japan) on an ABI QuantStudio6 Pro (Applied Biosystems USA), and gene expression levels were normalized to *GAPDH*. Three samples were extraction and independently performed three replicates of the qRT-PCR experiment.

### Western blotting

After 48 h of treatment with different concentrations of PD, HSFs were lysed in radioimmunoprecipitation assay (RIPA) buffer for 30 min on ice. After lysis was complete, the liquid was collected and centrifuged for 10 min. The supernatant was used to measure the protein concentration (BCA protein assay kit, Thermo Fisher Scientific, USA). Extracted protein (20 mg) was separated by 10% sodium dodecyl sulfate–polyacrylamide gel electrophoresis (SDS-PAGE) and transferred to polyvinylidene difluoride (PVDF) membranes (Millipore, USA). After being blocked with 5% bovine serum albumin (BSA), the membrane was incubated overnight at 4 °C with primary antibodies. The primary antibodies were anti-type I collagen (Col I), anti-α-SMA, anti-matrix metalloproteinase 2 (MMP2), anti-type III collagen (Col III), anti-TGF-β1, anti-TGF-β receptor I (TGFβ RI), anti-phospho-Smad2/3 (p-Smad2/3) and anti-Smad2/3 (all from Cell Signaling Technology, USA). The next day, the PVDF membrane was incubated with the corresponding horseradish peroxidase (HRP)-conjugated secondary antibodies (Cell Signaling Technology, USA) on a slow shaker at room temperature for 1 h. Then, the membrane was washed 3 times and analyzed with Odyssey V3.0 image scanning (Li-COR. Inc., Lincoln, NE, USA). β-actin (Cell Signaling Technology, USA) was used as a loading control.

### Cell counting kit-8 (CCK-8) assay

Briefly, HSFs were seeded in 96-well plates (3 × 10^4^/mL and 100 μl/well) and treated with 0, 2, 4, 6, 8 or 10 μM PD. Five replicate wells were used for each concentration. After cells adhered and stabilized, the corresponding concentration of PD was added for 0, 12, 24, 48 and 72 h. CCK-8 solution (10 μl; Dojindo, Japan) was then added to each well and incubated for 2 h at 37 °C in the dark. Cell viability was quantified by measuring the absorbance at 450 nm with a microplate reader (Biotek, Vermont, USA).

### Wound healing assay

HSFs were seeded in 6-well plates at a density of 5 × 10^5^/well in CM. When the cells reached confluence, the medium was replaced with DMEM containing 1% serum for 12 h. A scratch wound with a uniform width was made, and the floating cells were washed away with sterile phosphate-buffered saline (PBS). The HSFs in each well were treated with 0, 2, 4, 6 or 8 μM PD and cultured for 24 h. The size of the scratch was recorded with an inverted microscope (Nikon, Japan) at 0, 6, 12 and 24 h [39].

### Transwell assay

HSFs (1 × 10^5^/mL and 200 μl of DMEM) were added to the upper chamber of Millicell Hanging Cell Culture Inserts (Millipore, USA), and 700 μl of CM containing different concentrations of PD (0, 2, 4 or 8 μM) was added to the lower chamber. After 24 h of incubation, the chamber was removed and fixed with 4% paraformaldehyde for 5 min, gently washed 3 times and stained with crystal violet for 10 min. Cells in the upper layer of the chamber were gently wiped away with cotton swabs, and cells in the lower layer of the chamber were quantified by taking pictures under a microscope (Nikon, Japan).

### Annexin V/PI staining

After cells adhered and stabilized, they were treated with 0, 2, 4, 6 or 8 μM PD for 48 h. Cells were then digested with trypsin and washed with cold PBS. Apoptosis was detected using the Alexa Fluor^®^ 488 annexin V/dead cell apoptosis kit (Invitrogen, USA) and flow cytometry (Beckman, USA), and the results were analyzed by FlowJo software version 10 (FlowJo, LLC, Ashland, Oregon, USA).

### Immunofluorescence analysis

HSFs (1 × 10^5^/well) were seeded on cover slips and treated with PD (0 μM), TGF-β1 (5 ng/mL) or TGF-β1 (5 ng/mL) + PD (8 μM) for 48 h. Cells were then fixed with 4% paraformaldehyde for 15 min, permeabilized with 0.5% Triton X-100 (Sigma, USA) for 20 min and blocked with 5% goat serum (Gibco, USA) for 1 h. Slides were incubated with rabbit monoclonal anti-α-SMA antibodies (Abcam, UK) at 4 °C overnight, and on the next day, they were incubated with Alexa Fluor 488 goat anti-rabbit IgG secondary antibodies (Abcam, UK) for 1 h at room temperature. Slides were then sealed with DAPI Fluoromount-G (SouthernBiotech, USA), and a confocal microscope (Nikon, Japan) was used to analyze the fluorescence intensity.

### Animal model

Animal experiments and surgical operations were performed in accordance with the “Guide for Care of Laboratory Animals” outlined by the National Ministry of Science and were approved by the Animal Ethical Committee of Jiagan Biotechnology Co., Ltd (Approval number: JGLL-20200620). Twelve New Zealand white rabbits (6–8 weeks old and weighing 2.5–3.0 kg) were used, and all animals were raised and processed using standard procedures.

Anesthesia and analgesia were induced before the operation. Atropine sulfate (0.05 mg) was subcutaneously injected, and 5 mg/kg Zoletil^®^ 50 (Virbac SA) was injected into the ear vein 15 min later. The auxiliary analgesic and muscle relaxant, xylazine hydrochloride (1 mg/kg), was then injected intramuscularly. An 8 mm biopsy punch was used to establish four full-thickness dermal defects on the ventral sides of both ears, and the perichondrium was removed to completely expose the cartilage. At 24 h post-operation, each wound on the left ear of the rabbit was injected with normal saline as a control, while the right ear was injected with different concentrations of PD as follows: low concentration group (8 μM in 100 μl, *n* = 6) and high concentration group (40 μM in 100 μl, *n* = 6). The injections were performed in four equal locations around the wounds.

Animals were fed normally after the surgery. After 14 or 28 days of continuous PD administration, animals were sacrificed using air embolization under anesthesia state, and full-thickness scars were obtained. Calipers were used to measure the maximum protuberant heights of HSs and normal skin. Wounds were evaluated by the scar elevation index (SEI) [6], which is the ratio of the total thickness of the wound to that of normal tissue.

### Sirius red staining

After the samples were fixed with 4% paraformaldehyde for 24 h, they were rinsed with running water. After dehydration in graded ethanol, the paraffin-embedded tissue was cut into 5 μm-thick sections. Tissue sections were stained with Sirius red stain (Fluka, Switzerland) and observed with a polarized upright microscope (Nikon, Japan). ImageJ software was used to measure and analyze the collagen fibers.

### Immunohistochemistry

Immunohistochemistry (IHC) was performed to examine proliferating cell nuclear antigen (PCNA) and α-SMA levels. After deparaffinizing the section, pepsin (Sigma, USA) was used for antigen retrieval at 37 °C for 30 min. Then, 3% H_2_O_2_ was applied to block endogenous peroxidase activity. Mouse monoclonal anti-PCNA antibodies and anti-α-SMA antibodies (Abcam, UK) were added and incubated at 4 °C overnight. Sections were then incubated with goat anti-mouse IgG-HRP (Invitrogen, USA) for 1 h at room temperature. Finally, sections were stained with a DAB detection kit (Vector Laboratories, Burlingame, CA) and counterstained with hematoxylin.

### Statistical analysis

All data were presented by mean ± SD, and analyzed using SPSS version 20.0 (SPSS, Inc., Chicago, IL, USA). Model assumptions were evaluated by examining the residual plot. Measurement data between two groups were compared and analyzed using the paired t-test and that of intergroup comparison were analyzed using one-way ANOVA with Dunnett’s test. *P* < 0.05 was considered statically significant.

## Results

### PD reduces the mRNA and protein expression of Col I, Col III and α-SMA in HSFs

To study the inhibitory effects of PD on fibrosis and collagen deposition, HSFs were treated with 0, 2, 4, 6 or 8 μM PD for 3 days. The differences in fibrosis-related genes were analyzed by qRT-PCR and Western blotting, respectively. The qRT-PCR results showed that PD dose-dependently downregulated the mRNA levels of *Col I, Col III* and *α-SMA*, and the effect was significant at 6 or 8 μM PD (Fig. 1B–D) (*p* < 0.05). This trend was also verified by Western blotting (Fig. 1E). In response to 6 μM PD, the protein expression levels of Col I and Col III were significantly downregulated (Fig. 1F and G) (*p* < 0.05). However, the protein expression of α-SMA only showed a declining trend in response to 8 μM PD (Fig. 1H) (*p* < 0.05).

### PD inhibits HSFs proliferation and migration but induces HSFs apoptosis in vitro

**Fig. 2:**
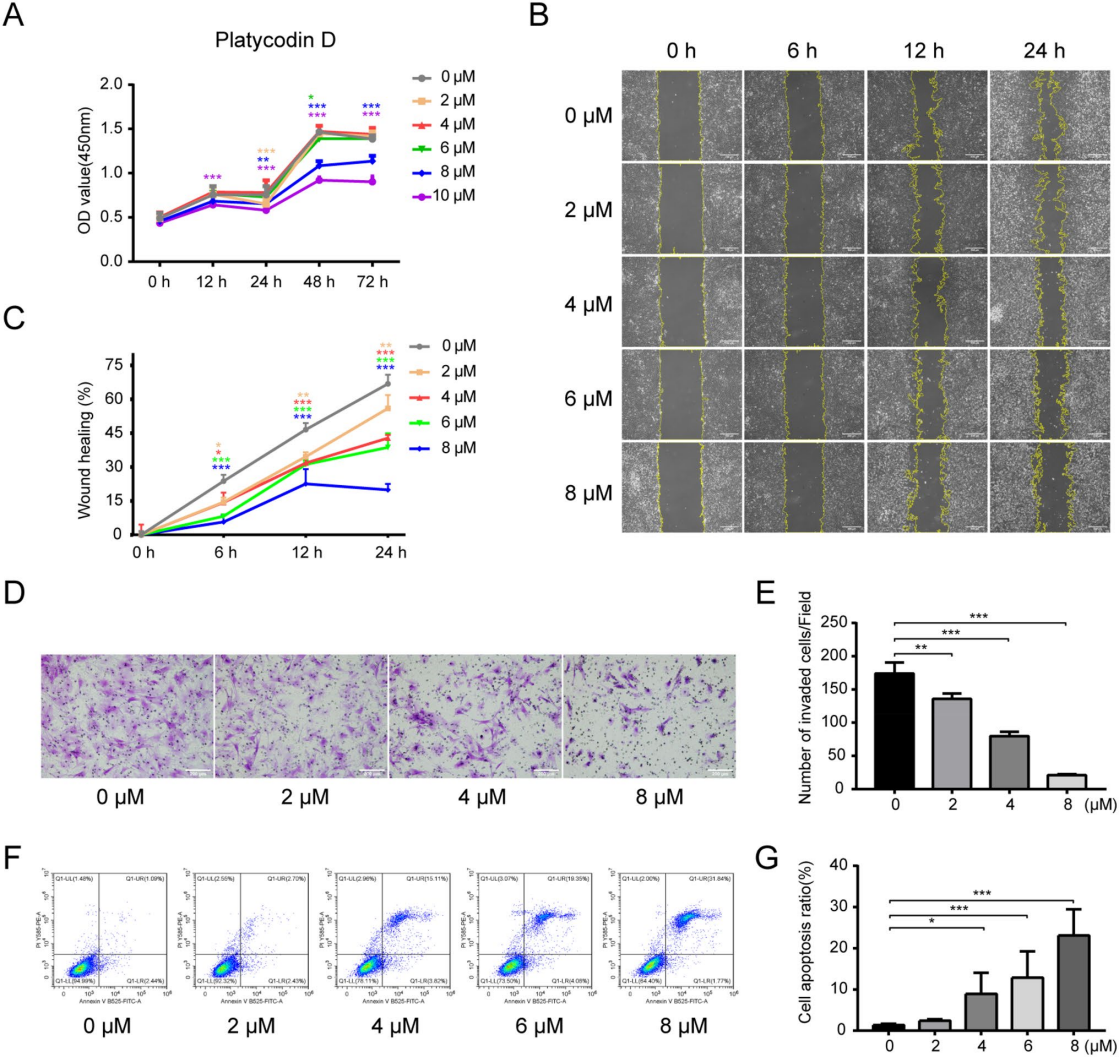
Effects of PD on HSFs proliferation, migration and apoptosis (**A**) Proliferation curves of HSFs after treatment with 0, 2, 4, 6 or 8 μM PD for 0, 12, 24, 48 and 72 h as determined by CCK-8 assays. **B** Representative images of the wound healing assay after treatment with 0, 2, 4, 6 or 8 μM PD for 0, 6, 12 and 24 h. The images were acquired using a Nikon phase-contrast microscope at 200× . Scale bar = 100 μm. **C** Quantification of the wound area in **B**. **D** Representative images of the Transwell assay after treatment with 0, 2, 4 or 8 μM PD for 24 h. Images were acquired using a Nikon microscope at 200× . Scale bar = 200 μm. **E** Quantification of the number of invaded HSFs per field in **D**. **F** Flow cytometry analysis of HSFs apoptosis after treatment with 0, 2, 4, 6 or 8 μM PD for 48 h. **G** Quantification of the cell apoptosis ratio in **F**. Data are presented as the mean ± SD. (*n* = 3); *h* hours; *p < 0.05, **p < 0.01 and ***p < 0.001

The effects of different concentrations of PD on HSFs were analyzed by CCK-8 assays. As shown by the cell proliferation curves, PD inhibited cell proliferation in a dose-dependent manner at concentrations ranging from 6 to 10 μM (Fig. 2A) (*p* < 0.05). Next, the HSF migration affected by PD were examined by wound healing and Transwell assays. The results of the wound healing assay showed that the migration of PD-treated HSFs was inhibited at 12 and 24 h (Fig. 2B). After quantitative analysis, the wound healing percentages in the PD-treated groups were significantly lower than those in the control group (Fig. 2C) (*p* < 0.05). The Transwell results indicated that the cell migration in the PD treatment group was decreased compared to that of the control group (Fig. 2D) as indicated by the quantitative analysis (Fig. 2E) (*p* < 0.05). Flow cytometric analysis showed that PD promoted HSFs apoptosis (Fig. 2F), and the proportion of apoptotic cells gradually increased from 2.7 to 31.84% with increasing concentrations of PD (Fig. 2G) (*p* < 0.05).

### PD inhibits the activation of α-SMA in HSFs

**Fig. 3:**
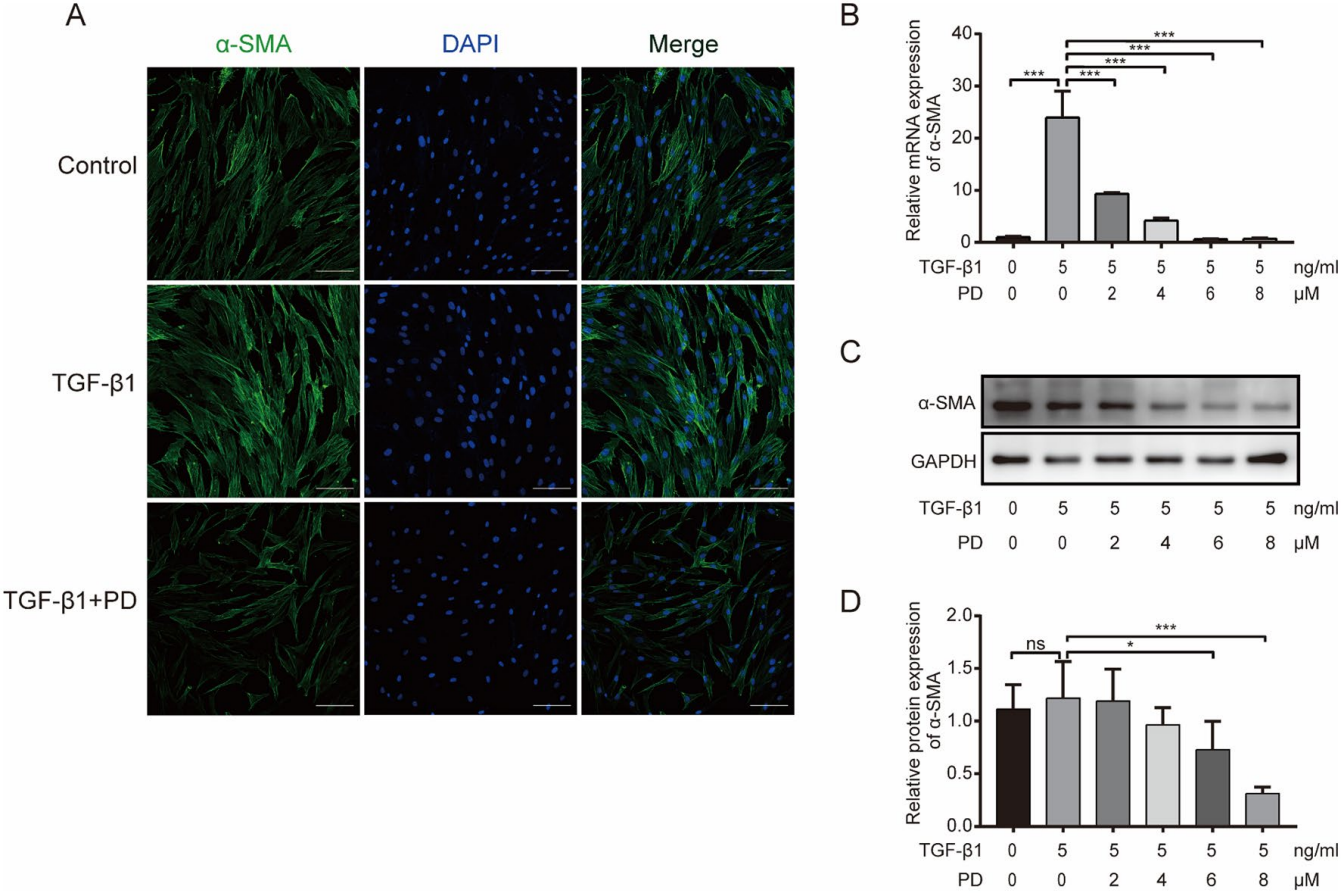
PD suppresses HSFs activation in vitro (**A**) Immunofluorescence staining of α-SMA in HSFs after treatment with PD (0 μM), TGF-β1 (5 ng/mL) and TGF-β1 (5 ng/mL) + PD (8 μM) for 48 h. α-SMA is shown by green fluorescence. Nuclei were stained with DAPI and are shown by blue fluorescence. The pictures were acquired using a Nikon confocal microscope at 200× . Scale bar = 100 μm. **B** qRT-PCR analysis of α-SMA mRNA expression in HSFs after treatment with PD (0 μM), TGF-β1 (5 ng/mL), TGF-β1 (5 ng/mL) + PD (2 μM), TGF-β1 (5 ng/mL) + PD (4 μM), TGF-β1 (5 ng/mL) + PD (6 μM) and TGF-β1 (5 ng/mL) + PD (8 μM). β-actin served as the control. **C** Western blot analysis of α-SMA protein expression in HSFs after treatment with PD (0 μM), TGF-β1 (5 ng/mL), TGF-β1 (5 ng/mL) + PD (2 μM), TGF-β1 (5 ng/mL) + PD (4 μM), TGF-β1 (5 ng/mL) + PD (6 μM) and TGF-β1 (5 ng/mL) + PD (8 μM). GAPDH was used as a loading control. **D** Quantification of the α-SMA protein level in **C**, which was normalized to the level of GAPDH. Data are presented as the mean ± SD. (*n* = 3); *p < 0.05, **p < 0.01 and ***p < 0.001

Studies have shown that HSs show relatively high expression of α-SMA; and TGF-β1 can induce α-SMA expression in HSs [19]. To study the effect of PD on the function of TGF-β1 in HSFs, cells were treated with PD (0 μM), TGF-β1 (5 ng/mL), TGF-β1 (5 ng/mL) + PD (2 μM), TGF-β1 (5 ng/mL) + PD (4 μM), TGF-β1 (5 ng/mL) + PD (6 μM), or TGF-β1 (5 ng/mL) + PD (8 μM), and the results showed that PD inhibited the effect of TGF-β1 on α-SMA activation. Immunofluorescence staining (Fig. 3A) showed that the expression of α-SMA induced by TGF-β1 was significantly reduced by high concentrations of PD. qRT-PCR and Western blotting (Fig. 3B and C) showed similar results, and the effects were most significant at 8 μM PD (Fig. 3D) (*p* < 0.05).

### PD alleviates hypertrophic scar formation in vivo

**Fig. 4:**
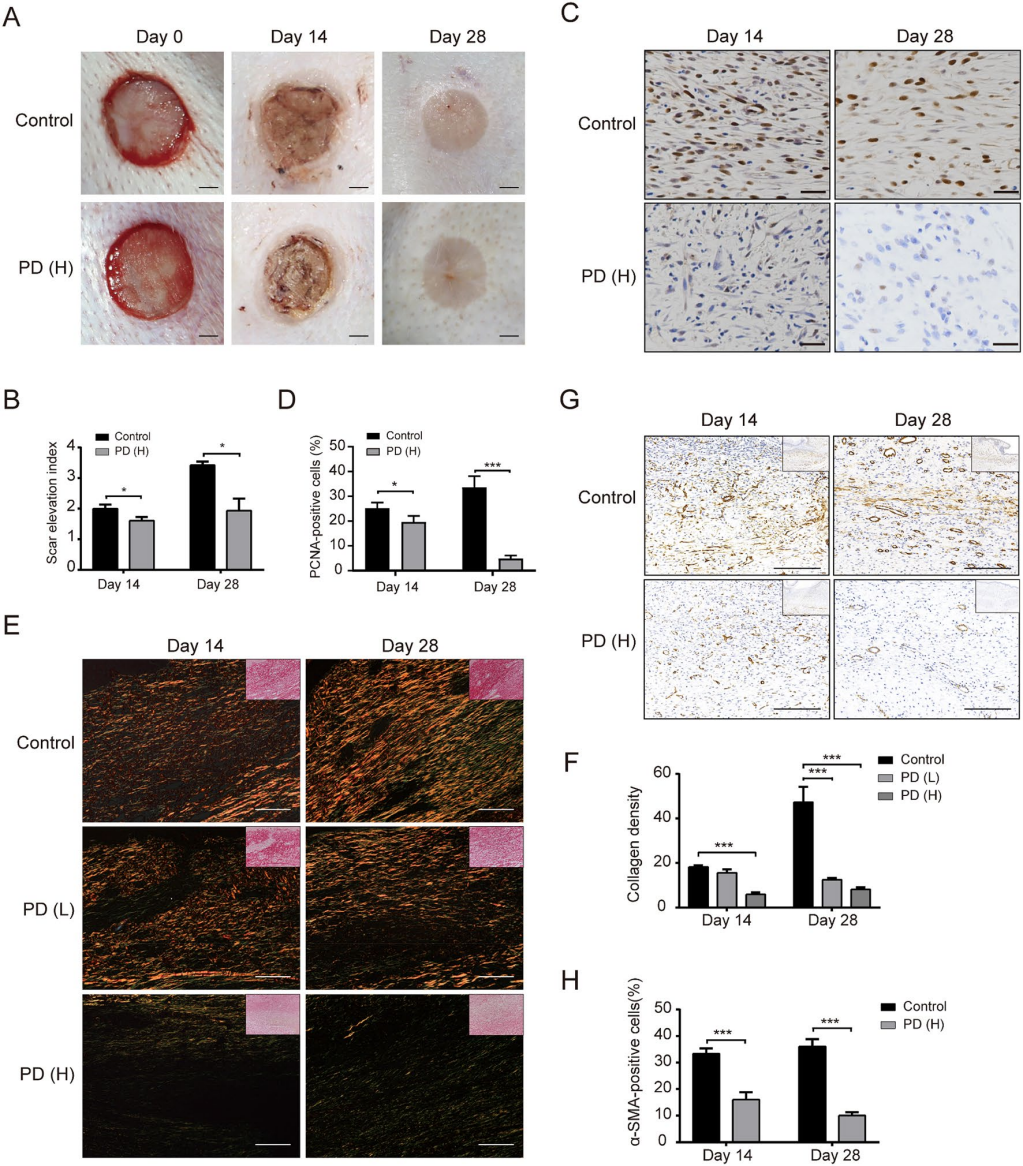
PD alleviates HS formation in a rabbit ear scar model (**A**) Representative images of rabbit ear scars at days 14 and 28 in the control group and the PD group. Scale bar = 10 mm. **B** SEI quantification of normal saline-treated scars and PD-treated scars (40 μM) 14 and 28 days after the operation. **C** IHC images of HS samples highlighting PCNA-positive cells after treatment with normal saline or PD (L = 8 μM and H = 40 μM) on days 14 and 28 (200× , scale bar = 100 μm). **D** Quantification of the percentage of PCNA-positive cells shown in **C**. **E** Sirius red staining of HS samples under polarized light from the normal saline or PD (L = 8 μM and H = 40 μM) groups on days 14 and 28 (200× , scale bar = 100 μm). **F** Quantification of the collagen density measured in **E**. **G** Representative images of immunohistochemical staining showing α-SMA-positive cells in the normal saline group and PD (*H* = 40 μM) group on days 14 and 28 (200× , scale bar = 200 μm). **H** Quantification of the percentage of α-SMA-positive cells shown in **G**. Data are presented as the mean ± SD. (*n* = 6); *p < 0.05, **p < 0.01 and ***p < 0.001

The effect of PD was evaluated on a rabbit ear scar model in vivo. The images of the scars and the SEI score were recorded on days 14 and 28 (Fig. 4A). The results showed that compared with the control group, scars treated with a high concentration of PD (40 μM) were alleviated, and the SEI was also significantly reduced (Fig. 4B) (*p* < 0.05). The number of PCNA-positive cells in the scar samples from the high concentration (40 μM) PD treatment group was significantly reduced on days 14 and 28 (Fig. 4C). A significant difference was observed after 28 days of treatment rather than 14 days (Fig. 4D) (*p* < 0.05). The Sirius red staining showed a distribution of Col I in the scar samples (Fig. 4E), which was significantly greater on day 28 compared to day 14. However, Col I was markedly inhibited after treatment with low and high concentrations of PD (Fig. 4F) (*p* < 0.05). Compared to the control group (Fig. 4G), IHC staining and quantitative analysis of α-SMA showed that HSFs proliferation and angiogenesis in the scar area were significantly decreased in the high concentration PD group (Fig. 4H) (*p* < 0.05).

### PD inhibits the expression of proliferation- and migration-related proteins in HSFs and induces apoptosis through a caspase-dependent pathway

**Fig. 5:**
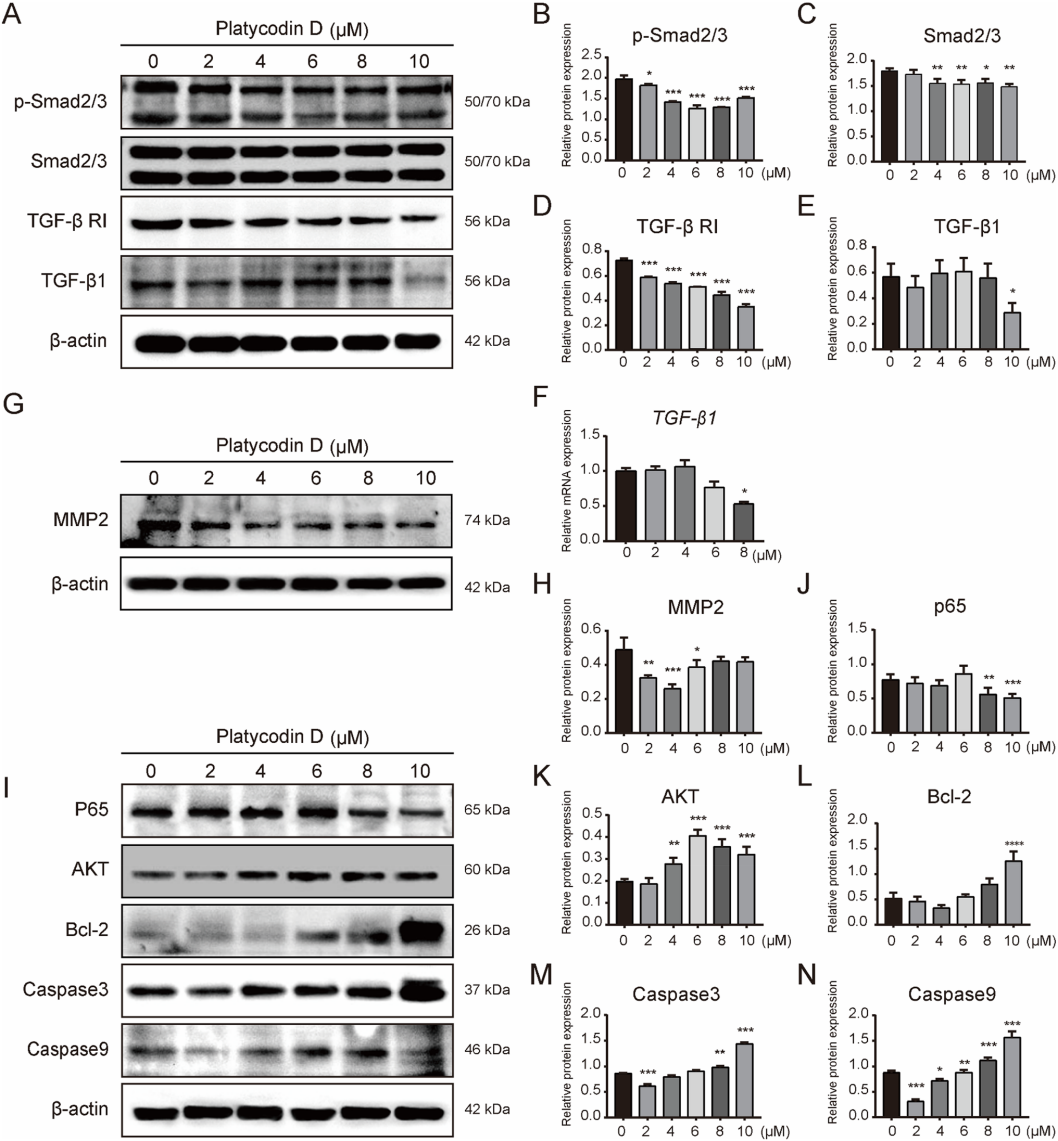
PD inhibits the expression of proliferation- and migration-related proteins in HSFs and induces apoptosis through a caspase-dependent pathway (**A**) PD treatment attenuated the expression of TGF-β RI and inhibited the activation of Smad2/3. HSFs were seeded at 1 × 10^5^ cells/well in 6-well plates and treated with PD (0, 2, 4, 6, 8 or 10 μM) for 48 h. Western blot analysis was performed with antibodies specific to p-Smad2/3, Smad2/3, TGF-β RI and TGF-β1. **B**–**E** Quantification of the protein level in (**A**). **F** PD treatment with 8 μM suppressed transcriptional expression of *TGF-β1*. HSFs were treated with PD (0, 2, 4, 6 or 8 μM) for 48 h. The expression of *TGF-β1* was normalized to GAPDH (*p* < 0.05). **G** PD treatment suppressed MMP2 expression. HSFs were treated with PD (0, 2, 4, 6 or 8 μM) for 48 h. Whole-cell lysates were collected for Western blotting analysis. **H** Quantification of the MMP2 protein level in **G**. **I** PD treatment promoted the activation of apoptotic proteins and inhibited the expression of p65. HSFs were treated with PD (0, 2, 4, 6, 8 or 10 μM) for 48 h. **J–N** Quantification of the protein level in **I**. *p < 0.05, **p < 0.01 and ***p < 0.001

To explore the molecular mechanism of the effects of PD on the proliferation, migration and apoptosis of HSFs, the expression of related proteins was analyzed. Firstly, PD inhibited the activation of the Smad2/3 pathway in HSFs (Fig. 5A). With increasing concentrations of PD, the phosphorylation of Smad2/3 gradually decreased (Fig. 5B–C) (*p* < 0.05), and the expression of TGF-β RI also decreased in a dose-dependent manner (Fig. 5D) (*p* < 0.05). However, at high concentration (10 μM) of PD, the protein expression of TGF-β1 was inhibited significantly (Fig. 5E) (*p* < 0.05). In addition, the mRNA expression of *TGF-β1* increased slightly at low PD concentration and decreased at high PD concentration (*p* < 0.05), which was consistent with the protein expression of TGF-β1 (Fig. 5F). Moreover, PD inhibited the protein expression of MMP2 (Fig. 5G), which is an important protein related to cell migration, but the inhibition of MMP2 did not change significantly with the increase of PD concentration (Fig. 5H) (*p* < 0.05). Then the protein expression of caspase 3, caspase 9 and Bcl-2, which are important proteins related to cell apoptosis, were analyzed (Fig. 5I). The result showed that the protein expression of caspase 3 and caspase 9 increased slowly with increasing concentrations of PD (Fig. 5M–N) (*p* < 0.05). Interestingly, barely detectable levels of Bcl-2 were found at low PD concentration, but these levels increased significantly at high PD concentration (Fig. 5L) (*p* < 0.05). At the same time, the expression of p65 decreased at high PD concentration (Fig. 5J) (*p* < 0.05), but the expression of AKT was not affected (Fig. 5K).

## Discussion

HSs, as an abnormal result of scar repairing, are characterized by excessive fibrosis and massive collagen deposition [1, 4]. However, the existing treatment methods are neither effective nor ideal. In the pathological process of HSs formation, fibroblasts are activated, and ECM (such as Col I and Col III) is deposited in large amounts [2, 10]. In this study, both in vitro and in vivo experiments were used to evaluate the biological function of PD on HSFs and to explore the role of PD on HSs in animals. The results showed that PD not only inhibited collagen deposition but also inhibited the proliferation and migration of HSFs and promoted cell apoptosis, which has potential application value in the treatment of HSs. In our study, PD significantly reduced the expression of Col I, Col III and α-SMA at the mRNA and protein levels in a dose-dependent manner, which is consistent with reversing the fibrosis of myocardium and liver cells [20, 22]. In addition, PD significantly inhibited the expression of TGF-β RI, but the effect of PD on the protein expression of TGF-β and total Smad2/3 was not obvious. However, PD decreased p-Smad2/3 expression, indicating that the inhibitory effect of PD on fibrosis is achieved by inhibiting the TGF-β/Smad pathway.

According to previous studies, TGF-β binds to TGF-β RII to form phosphorylated TGF-β RI, which phosphorylates Smad2/3 and, in turn, affects the proliferation and differentiation of HSFs [5, 24, 38]. Blockade of the TGF-β1/Smad pathway inhibits the proliferation of fibroblasts and the synthesis of collagen, thereby reducing the process of HSs [17, 36]. Although the total Smad and TGF-β protein expression levels did not change significantly in our study, the p-Smad protein content decreased at high PD concentrations, and the TGF-β RI expression level was significantly decreased after PD treatment. Our results suggested that PD inhibits the progression of HSs by inhibiting TGF-β RI protein expression. Thus, TGFβ RI may be a target of PD and will be studied in the future. Previous studies have shown that PD reduces the expression of MMP2 and MMP9 to inhibit the invasion and metastasis of human oral squamous cell carcinoma [37]. Our results showed that PD inhibited the expression of MMP2 in HSFs. These findings suggested that PD alleviates the invasion of HSFs into the scar by regulating the MMP-mediated degradation pathway of ECM.

The formation of HSs is strongly related to insufficient apoptosis of fibroblasts [11]. Failure to activate caspase proteins in HSFs leads to insensitivity of cells to the apoptotic response and an enhanced anti-apoptotic response mediated by TGF-β1 [8]. Moreover, the expression level of Bcl-2 in HSFs is higher than that of normal fibroblasts [21]. Studies have shown that PD induces apoptosis in liver cancer cells [15], breast cancer cells [34] and non-small cell lung cancer [27]. Thus, previous studies have considered PD as a potential anti-tumor therapeutic drug, which exerts an anti-cancer effect by inhibiting angiogenesis, promoting cell apoptosis and inhibiting the cell cycle. In human HSFs, however, it remains unknown whether PD affects the proliferation and apoptosis of fibroblasts. The present study suggested that PD promotes the apoptosis of fibroblasts.

The caspase family is a protease family that plays an important role in cell apoptosis, including apoptotic initiators (caspases 2, 8, 9 and 10) and executors (caspases 3, 6 and 7) [12]. According to our results, after 2 days of PD treatment of HSFs, the protein levels of the caspase 3 and caspase 9 apoptotic factors increased significantly, indicating that PD may promote apoptosis through a caspase-dependent pathway. In our research, PD inhibited Bcl-2 expression at a low concentration, indicating that PD attenuates the apoptosis resistance of HSFs within the effective concentration range. In addition, previous studies have shown that PD promotes the process of apoptosis by inhibiting the activation of the PI3K/AKT pathway [27, 31] and interfering with cell function by inhibiting NF-κb activation. Analysis of the total protein levels of AKT and p65 in HSFs after PD treatment showed that the total protein expression of AKT increased slightly, which was inconsistent with previous results. The results also showed that the total protein level of p65 decreased, indicating that PD inhibits the anti-apoptotic pathway mediated by NF-κb. The specific mechanism needs to be explored in the future.

## Conclusions

In summary, this study demonstrated that PD significantly inhibited the proliferation and migration of HSFs as well as promoted cell apoptosis. Further, PD significantly inhibited collagen synthesis and deposition of HSs in vivo. Our study also elucidated the mechanism of PD inhibiting fibroblast fibrosis and promoting apoptosis. Commercial compounds treating HSs are general expensive and may have side effects on human body. PD, as a common and traditional triterpenoid monomer compound, is cost-effective and has the strength of abundant supply. However, our study only conducted the in vivo experiment on rabbits. It is still necessary to verify the effect of PD in large animals, like pigs, to meet the needs of clinical translation. Based on these studies, our study suggest that PD may be a promising and effective agent to treat HSs and other fibrosis-related diseases.

## Data Availability

The datasets generated during the current study are available from the corresponding author on reasonable request.
